# Hardening Dental Rubbers in Steatite without the Aid of Flasks

**Published:** 1884-02

**Authors:** G. Brunton

**Affiliations:** Leeds.


ARTICLE VII.

### HARDENING DENTAL RUBBERS IN STEATITE WITHOUT THE AID OF FLASKS.

BY G. BRUNTON, LEEDS.

(Paper read at the Annual Meeting of the British Dental Association, Plymouth, August 24th, 1883,

Every practitioner will admit that the use of the flask for packing dental rubbers is not a perfect one; that there are faults in the process, which produce in some cases porosity, in some raised bite, teeth shifted in position, etc., etc., faults which are exceedingly annoying to the workman and disappointing to the patient.

The process which I will try to describe to you is very simple and requires no special machinery for its performance; is applicable to all kinds of vulcanite work, both full and partial cases, combination work of gold and vulcanite, regulation plates, repairs in fact any case, no matter how complicated, can be done quicker and better by this method than by flasking in Plaster of Paris, for it is a well known fact that rubber is deteriorated by being hardened in plaster. The rubber which I prefer to use is bowspring. Owing to its superior softness before vulcanizing, it can be manipulated like wax. The models used are ordinary plaster ones, but to prevent contact with the rubber they are coated with liquid silex, this fills the pores of the plaster, produces a smooth polished surface on the palatial side of the finished work, and prevents the steam from exerting pressure on the rubber where it is not wanted, namely, on the palatial side. The models being dry are coated with a thin solution of rubber in chloroform, and a piece of sheet rubber of the desired size and shape is warmed and pressed close to the model, excluding all air. Any extra thickness may be built up by adding small pieces of warm rubber. The teeth to be mounted are now put on a small metal tray and heated over a spirit lamp, and one by one placed in position on the model. When all the teeth are mounted, trim off the extra rubber with a hot knife, cover the rubber with tin foil, taking care not to disturb the position of the teeth. The work is now ready for hardening, and is placed in a tin box which is large enough to allow about ¾ inch room all around for the investment; this consists of steatite or soap-stone in powder. The dry powder is pressed lightly in till the box is quite full, the lid is closed and fastened down with a thin piece of wire. The hardening may be done in any vulcanizer, but I prefer to use Dr. Campbell's new mode heater, as it can be brought to the proper temperature before putting in the work. The steam pressure is brought to 60 lbs., and after the door is screwed on a very small quantity of steam is let into the hot box and left for three hours. When ready to remove, the tin box may be opened while hot. The steatite shakes out quite dry, and may be used over again many hundreds of times. The work is now to be cooled in water, the tin foil removed and the case finished in the ordinary way. Dental rubber so treated will be found to possess more toughness and density than that which is hardened in Plaster of Paris. The sp. gr. of bowspring rubber is before vulcanizing 1.4632; after vulcanizing in Plaster of Paris it is 1.5103; and after vulcanizing in steatite 1.5278. This you will see shows greater density in rubber hardened in steatite. A proof of the superiority of this process is found in the close adaptation of the rubber to the teeth, there is no space left for food to lodge in. To show you how little risk there is of porosity, I hand around a cube of rubber one inch square, which was hardened with a steam pressure of 45 lbs., time four hours.

In conclusion, I would refer any who wish for fuller information on the subject of producing rubber work without the aid of flasks to Mr. Balkwell's book on Mechanical

Dentistry, from which I first got the idea. *All* my rubber work has been done for some years without the aid of flasks, and I hope the communication of this method may prove of as much benefit to those who may try it, as it has been to me.—*British Journal of Dental Science.*

---

### EDITORIAL, ETC.

